# High-Level Macrolide Resistance Due to the Mega Element [*mef*(E)/*mel*] in *Streptococcus pneumoniae*

**DOI:** 10.3389/fmicb.2019.00868

**Published:** 2019-04-24

**Authors:** Max R. Schroeder, Sarah Lohsen, Scott T. Chancey, David S. Stephens

**Affiliations:** ^1^Department of Medicine, Emory University School of Medicine, Atlanta, GA, United States; ^2^Laboratories of Microbial Pathogenesis, Department of Veterans Affairs Medical Center, Atlanta, GA, United States

**Keywords:** *Streptococcus pneumoniae*, pneumococcus, macrolide resistance, Mega, *mef*(E)/*mel*, *erm*(B), Tn*2010*

## Abstract

Transferable genetic elements conferring macrolide resistance in *Streptococcus pneumoniae* can encode the efflux pump and ribosomal protection protein, *mef*(E)/*mel*, in an operon of the macrolide efflux genetic assembly (Mega) element- or induce ribosomal methylation through a methyltransferase encoded by *erm*(B). During the past 30 years, strains that contain Mega or *erm*(B) or both elements on Tn*2010* and other Tn*916*-like composite mobile genetic elements have emerged and expanded globally. In this study, we identify and define pneumococcal isolates with unusually high-level macrolide resistance (MICs > 16 μg/ml) due to the presence of the Mega element [*mef*(E)/*mel*] alone. High-level resistance due to *mef*(E)/*mel* was associated with at least two specific genomic insertions of the Mega element, designated Mega-2.IVa and Mega-2.IVc. Genome analyses revealed that these strains do not possess *erm*(B) or known ribosomal mutations. Deletion of *mef*(E)/*mel* in these isolates eliminated macrolide resistance. We also found that Mef(E) and Mel of Tn*2010*-containing pneumococci were functional but the high-level of macrolide resistance was due to Erm(B). Using *in vitro* competition experiments in the presence of macrolides, high-level macrolide-resistant *S. pneumoniae* conferred by either Mega-2.IVa or *erm*(B), had a growth fitness advantage over the lower-level, *mef*(E)/*mel*-mediated macrolide-resistant *S. pneumoniae* phenotypes. These data indicate the ability of *S. pneumoniae* to generate high-level macrolide resistance by macrolide efflux/ribosomal protection [Mef(E)/Mel] and that high-level resistance regardless of mechanism provides a fitness advantage in the presence of macrolides.

## Introduction

*Streptococcus pneumoniae*, the pneumococcus, is an obligate commensal of the human nasopharynx and a worldwide opportunistic pathogen. *S. pneumoniae* causes non-invasive diseases such as acute otitis media, sinusitis, and pneumonia, as well as invasive diseases such as sepsis and meningitis (O’Brien et al., 2009). Antibiotic therapy for community-associated upper respiratory tract bacterial infections where *S. pneumoniae* is suspected often includes a macrolide (Suda et al., 2014; Hicks et al., 2015). In the United States, macrolides are one of the most prescribed antibiotics with 190 prescriptions per 1000 people in 2011 (Hicks et al., 2015). However, macrolide effectiveness has been compromised by the emergence of macrolide resistance in *S. pneumoniae* in the early to mid-1990s (Gay et al., 2000; Hyde et al., 2001). The continuing widespread use of macrolides has resulted in a strong selective pressure contributing to the expansion of macrolide-resistant *S. pneumoniae* (Appelbaum, 2002). While the pneumococcal conjugate vaccines (PCV7, PCV13), introduced in the US in 2000 and 2010, respectively, have significantly reduced the burden of pneumococcal disease as well as the overall incidence of antibiotic resistance in pneumococci, the strong selection pressure for resistance continues in the population.

Macrolide resistance in *S. pneumoniae* is predominantly due to ribosomal modification or macrolide efflux/ribosomal protection (Roberts et al., 1999; Schroeder and Stephens, 2016; Murina et al., 2018; Su et al., 2018). Macrolides bind to the 23S rRNA (predominantly at residue A2058 for *Escherichia coli*) of the 50S ribosome to inhibit protein synthesis (Weisblum, 1995b). A ribosomal methyltransferase, encoded by *erm*(B), prevents binding of macrolides by dimethylating the target site on the ribosome (Weisblum, 1995a). Ribosomal methylation results in high-level macrolide resistance (erythromycin MIC > 256 μg/ml) as well as resistance to lincosamides and streptogramin B (the MLS_B_ phenotype). *erm*(B) is carried on a group of mobile genetic elements including the *erm*(B) element and complex elements including the *erm*(B) element inserted into Tn*916* (Tn*6002);* or *erm*(B) is carried on Tn*917* that can be inserted into Tn*916* (Tn*3872*) (Brenciani et al., 2007; Chancey et al., 2015a).

Macrolide efflux/ribosomal protection is carried on the Macrolide Efflux Genetic Assembly (Mega), a 5.5 kb (Mega-1), or 5.4 kb (Mega-2) genetic element carrying *mef*(E) and *mel*, encoding a proton motive force efflux pump and a ribosomal protection protein (Tait-Kamradt et al., 1997; Gay and Stephens, 2001; Schroeder and Stephens, 2016). Mega is found in at least five locations in the pneumococcal genome (most common insertion classes being I, II, III, and IV) and also can be a component of larger genetic elements (e.g., insertion class V) (Gay and Stephens, 2001; Del Grosso et al., 2004; Chancey et al., 2015a). The *mef*(E)/*mel* operon confers resistance to 14- and 15-membered macrolides only (the M phenotype) and is inducible by these same macrolide molecules (Ambrose et al., 2005; Chancey et al., 2011). In the United States, most pneumococcal macrolide resistance has been due to *mef*(E)/*mel* (Stephens et al., 2005; Rudolph et al., 2013; Schroeder and Stephens, 2016). Mega-mediated macrolide resistance has been generally reported as MICs of erythromycin as 1–16 μg/ml (Ambrose et al., 2005; Chancey et al., 2012), and the clinical significance of Mega-mediated macrolide resistance has been debated (Cilloniz et al., 2015).

*S. pneumoniae* isolates with both macrolide resistance determinants, *erm*(B) and Mega [*mef*(E)/*mel*], have been identified (Corso et al., 1998; Luna et al., 1999; Nishijima et al., 1999; McGee et al., 2001; Del Grosso et al., 2006; Rudolph et al., 2013). In Atlanta, we documented the emergence and clonal expansion of macrolide-resistant serotype 19A clonal complex 320 isolates that contain Tn*2010*, containing both the *erm*(B) element in *orf*20 and Mega in *orf*6 of Tn*916* (Chancey et al., 2015a; Schroeder et al., 2017).

In this report, new high-level macrolide resistance of *S. pneumoniae* (MIC ≥ 16–256 μg/ml) due to the Mega element alone was identified and the genetic basis investigated. We also assessed the contribution of *erm*(B) and *mef*(E)/*mel* in the Tn*2010* dual element resistance isolates. Further, we found high-level macrolide resistance, regardless of mechanism, provided a competitive growth advantage during exposure to erythromycin.

## Materials and Methods

### Bacterial Strains, Media, and Growth Conditions

The characteristics of *S. pneumoniae* strains used are listed in Tables 1, 2. A panel of 44 epidemiologically distinct, well-characterized Mega containing macrolide-resistant clinical isolates was investigated (Table 1). The *S. pneumoniae* isolates in this panel were selected from over 13,000 *S. pneumoniae* isolates identified through active prospective population-based surveillance of invasive pneumococcal disease in Atlanta between 1994 and 2011 (Schuchat et al., 2001; Chancey et al., 2015a). The isolates selected all contained the Mega element [*mef*(E)/*mel*] identified by PCR and/or nucleotide sequencing, and were chosen to evaluate resistance profiles based on the different locations of each Mega insertion site (Chancey et al., 2015a). Where possible, five strains for each insertion site were evaluated. Isolates were preferentially selected for which whole genome sequence data was available, and reflected a variety of capsule serotypes, antibiotic resistance phenotypes, and dates of isolation (Chancey et al., 2015a). For experiments, all *S. pneumoniae* strains were routinely grown on trypticase soy agar II containing 5% sheep’s blood (blood agar) or in Todd-Hewitt broth containing 0.5% yeast extract (THY). Plate cultures were grown at 37°C with 5% CO_2_ and broth cultures were grown in a 37°C water bath.

**Table 1:** *S. pneumoniae* isolates (including isolation year, serotype, MLST, clonal complex, and source) with Mega (genetic insertion site), *erm*(B), or both, and minimum inhibitory concentrations (MICs) to erythromycin.

Mega	*erm*(B)	Strain	MIC^a^	iMIC^a,b^	Isolation year	Serotype	MLST	Clonal complex	Source
Mega-1.I	None	GA17328	4	24	2000	6A	376	CC2090	Chancey et al., 2015a
		GA17457	8	48	2000	19A	199	CC199	Zähner et al., 2010; Chancey et al., 2015a
		GA16857	4–6	24–32	2002	6A	376	CC2090	GAEIP
		GA41348	6–8	32	2004	19A	199	CC199	GAEIP
		GA41437	3	24	2004	6A	376	CC2090	Chancey et al., 2015a
		GA41502	4	32	2004	19A	199	CC199	GAEIP
Mega-1.II	None	EU-NP04	4	16–24	2009	6C	2705	CC1379	Chancey et al., 2015a
		GA47033	4–6	16–24	2005	6C	4150	CC1379	Chancey et al., 2015a
		GA52306	4	12–24	2007	6C	3676	CC1379	Chancey et al., 2015a
		GA60190	8	16	2010	6C	1292	CC1379	Chancey et al., 2015a
Mega-2.II	None	GA11757	16	48	2000	14	13	CC15	GAEIP
		GA16531	8	48	2001	6B	146	CC156	Chancey et al., 2015a
		GA17530	16	48	2000	14	81slv	–	GAEIP
		GA41538	16	64	2004	6A	384	CC156	Chancey et al., 2015a
		GA41688	16	48	2004	14	13	CC15	Chancey et al., 2015a
		GA62371	24	96	2011	35B	–	–	GAEIP
		GA64571	32	48	2012	35B	–	–	GAEIP
		GA67281	64	> 256	2012	23A	–	–	GAEIP
		GA71819	48–64	> 256	2013	23A	–	–	GAEIP
		GA71862	32	96	2013	35B	–	–	GAEIP
Mega-1.III	None	GA17301	8	48	2000	9V	156	CC156	Chancey et al., 2015a
		GA17570	6	48	2001	9V	156	CC156	Chancey et al., 2015a
		GA18641	8–12	48–64	2002	9V	156	CC156	GAEIP
		GA41277	12–24	64	2004	19A	199	CC199	Chancey et al., 2015a
		GA47760	6–8	32	2006	11A	62	CC62	Chancey et al., 2015a
		GA62681	6–8	64	2011	15C	199	CC199	Chancey et al., 2015a
Mega-2.IVa	None	GA04375	18	96	1995	19F	236	CC320	Chancey et al., 2015a
		GA14846	64	> 256	2000	6B	1536	CC1536	GAEIP
		GA16242	64	> 256	2001	6B	1536	CC1536	Chancey et al., 2015a
		GA16374	64	> 256	2001	6B	1536	CC1536	GAEIP
Mega-2.IVc	None	GA17545	64	> 256	2000	6B	1536slv	CC1536	Chancey et al., 2015a,b
Mega-1.IVb	None	GA17828	16	64	2001	33F	2705	CC100	GAEIP
		GA19795	4	24	2004	33F	2705	CC100	GAEIP
		GA40189	2–3	24	2002	33F	2705	CC100	GAEIP
		GA41317	8	24–32	2004	33F	2705	CC100	Chancey et al., 2015a
		GA41318	8	32	2004	33F	2705	CC100	GAEIP
Mega-1.V Tn*2009*	None	GA16833	4	32–48	2002	19F	5053	CC320	Chancey et al., 2015a
		GA17227	8–12	24	2000	23F	242	CC242	Chancey et al., 2015a
		GA17371	12	96	2000	19F	8014	CC320	Chancey et al., 2015a
		GA41301	12	32	2004	23F	242	CC242	Chancey et al., 2015a
		GA41565	3–4	32	2004	19A	81	CC81	Chancey et al., 2015a,b
Mega-1.VI	None	GA02254	3–4	16	1994	14	124	CC156	Chancey et al., 2015a,b
	Tn*3872*	GA47597	> 256	>256	2006	3	180	CC180	Chancey et al., 2015a
	Tn*6002*	GA44194	> 256	>256	2005	19A	2543	CC63	Chancey et al., 2015a
Mega-2.V Tn*2010*	Tn*2010*	GA11856	> 256	>256	2000	19F	271	CC320	Chancey et al., 2015a
		GA16121	> 256	>256	2000	19F	236	CC320	Chancey et al., 2015a
		GA44288	> 256	>256	2005	19A	320	CC320	Chancey et al., 2015a
		GA47688	> 256	>256	2006	19A	320	CC320	Chancey et al., 2015a
		GA47778	> 256	>256	2006	19A	320	CC320	Chancey et al., 2015a

*^*a*^MICs determined by Etest in at least duplicate and reported as μg/ml. Ranges provided when replicates varied, and each range is within a twofold dilution. ^*b*^Subinhibitory concentration used for induction: 0.1 μg/ml.*

**Table 2:** Erythromycin minimum inhibitory concentrations (MICs) for *S. pneumoniae* strains and mutants [Mega insertion, serotype, MLST (clonal complex)] used in this study.

Strain	Uninduced MIC^a^	Induced MIC^a,b^	Relevant genotype	References
GA44288	>256	>256	Mega-1.V Tn*2010*, 19A, ST320 (CC320)	Chancey et al., 2015a
MS32	8	64	GA44288 Δ*erm*(B)::*aphA-3*	This study
MS41	>256	>256	GA44288 Δ*mef*(E)/*mel*::*cat*	This study
MS42	0.125^S^	^∗^	GA44288 Δ*erm*(B)::*aphA-3*, Δ*mef*(E)/*mel*::*cat*	This study
GA16242	64	>256	Mega-2.IVa, 6B, ST1536 (CC1536)	Chancey et al., 2015a
TS9001-3	0.125^S^	^∗^	GA16242 Δ*mef*(E)/*mel*::*aphA-3*	This study
GA17545	96	>256	Mega-2.IVc 6B, ST1536slv (CC1536)	Chancey et al., 2015b
XZ8012-5	0.19^S^	^∗^	GA17545 Δ*mef*(E)/*mel*::*aphA-3*	This study
NP112	0.19^S^	^∗^	no macrolide resistance genes, 6B, ST1536 (CC1536)	Chancey et al., 2015a
MS23	32	>256	NP112 +Mega-2.IVa	This study
MS30	0.19^S^	^∗^	MS23 Δ*mef*(E)/*mel*::*aphA-3*	This study
GA17457	8	64	Mega-1.I, 19A, ST199 (CC199)	Zähner et al., 2010
XZ8009	0.125^S^	^∗^	GA17457 Δ*mef*(E)/*mel*::*aphA-3*	Zähner et al., 2010
MS27	32	>256	XZ8009 +Mega-2-IVa	This study

*^*a*^MICs reported as μg/ml. ^*b*^Subinhibitory concentration used for induction: 0.5 μg/ml. ^*S*^Susceptible to erythromycin when MIC ≤ 0.5 μg/ml. ^∗^Susceptible strains were not tested for inducible MIC.*

### Antibiotic Susceptibility

To determine macrolide MICs, bacterial cultures were grown overnight on blood agar and subcultured onto blood agar or blood agar with 0.1 μg/ml erythromycin supplementation to induce resistance expression as a standard protocol in our laboratory (Zähner et al., 2010; Chancey et al., 2011). These overnight cultures were suspended to a density approximately equal to a 0.5 McFarland standard and streaked onto Mueller-Hinton agar containing 5% sheep’s blood. Erythromycin susceptibility tests were performed by applying an erythromycin Etest strip (bioMérieux). After an overnight incubation, erythromycin susceptibility was measured. Uninduced MICs of 1–16 μg/ml were classified as resistant and those >16 μg/ml were classified as high-level resistant.

### General DNA Manipulation and Transformations

To evaluate the basis for high level macrolide resistance in *S. pneumoniae*, a series of mutants were created (Table 2). Primer sequences are listed in Supplemental Table A1. PfuUltra II Fusion DNA polymerase (Agilent Technologies) or Q5 polymerase (New England Biolabs), restriction enzymes (New England Biolabs) and T4 DNA ligase (Invitrogen) were used for mutational cassette construction. Taq DNA polymerase (Applied Biosystems) or One Taq DNA polymerase (New England Biolabs) were used for screening putative mutants.

*S. pneumoniae* was transformed by a standard method that utilized the competence-stimulating peptide 1 (CSP-1) for induction of competence (Havarstein et al., 1995). CSP-1 was synthesized by the Emory University Microchemical Facility. Transformations were performed using plasmid DNA or PCR products and selected on blood agar containing kanamycin at 400 μg/ml, erythromycin at 1 μg/ml, or chloramphenicol at 3.2 μg/ml as described below. For mutants TS9001-3 and XZ8012-5, competent cells were transformed with a previously created plasmid that replaces *mef*(E) and *mel* with an *aphA-3* cassette and double crossover mutants were selected on kanamycin and confirmed by PCR and sequencing (Zähner et al., 2010). This method was also used to delete *mef*(E)/*mel* from GA16242 to create TS9001-3 and from GA17545 to create XZ8012-5 (Table 2).

To generate mutants MS23 and MS27, a 10.9 kb PCR product containing Mega-2.IVa was amplified using primers SC173 and SC251 and purified using the QIAquick Gel Extraction kit. Purified PCR products were transformed into NP112 to create MS23 and XZ8009 to create MS27 and transformants were selected on erythromycin. Insertions were confirmed by PCR of the left and right junctions of Mega-2 in insertion site IVa with primers SC10 with SC173 and SC70 with SC251 (Table 2). For mutant MS30, MS23 was transformed with BamHI digested *mef*(E)/*mel*::aphA-3 plasmid. The desired double crossover was selected on kanamycin. The insertion was confirmed by PCR amplification of a 1521 bp product with primers SC125 and kanA (Table 2).

To generate mutant MS32, GA44288 genomic DNA was amplified upstream (primers MS34 and MS35) and downstream (primers MS36 and MS37), and regions of *erm*(B) were spliced by overlapping extension (SOE) to create an internal XbaI site using the PCR amplified regions with primers MS34 and MS37. The resulting 1066 bp product was digested with BamHI and PstI and cloned into double-digested pUC19 vector to create pMRS11. The kanamycin resistance cassette, *aphA-3* was PCR amplified from pSF151 (Tao et al., 1992) using primers MS53 and MS54 and the product was XbaI digested and cloned into pMRS11 to create pMRS13. The *aphA-3* cassette was confirmed to be in the forward direction by PCR with primers MS34 and MS54. Transformation of GA44288 cells with pMRS13 and selection on kanamycin to created strain MS32, which was confirmed by PCR amplification with primers MS27 and kanA as well as MS28 and kanC (Table 2).

Finally, to generate mutants MS41 and MS42, PCR amplification of the *mef*(E) upstream region by primers MS64 and MS72, the *mel* downstream region by primers MS63 and MS69, and the chloramphenicol cassette from pEVP3 (Claverys et al., 1995) by primers MS70 and MS71 was performed. A single SOE PCR reaction with the three PCR products and primers MS63 and MS64 created a 2 kb Δ*mef*(E)/*mel*::cm^R^ cassette. This product was used for transformation and selection on chloramphenicol for GA44288 to create MS41 and MS32 to create MS42, which were confirmed by PCR amplification of a 2 kb product from primers MS63 and MS64 (Table 2).

### qRT-PCR

To determine *mef*(E)/*mel* expression, overnight blood agar cultures were first suspended in THY and grown to mid-log phase (OD_600_ = 0.3–0.5). Each culture was diluted to OD_600_ = 0.05 in prewarmed THY, grown to mid-log phase, and cultures were divided into tubes with or without erythromycin as indicated and continued to grow until the indicated treatment time was achieved. Culture aliquots were mixed with RNAprotect Bacterial Reagent (Qiagen) and RNA was isolated using the RNeasy Mini Kit (Qiagen). DNA was removed via the TURBO DNA-free (Applied Biosystems) and confirmed to be free of DNA by PCR using primers for genes of interest (Supplemental Table A1). QuantiTect Reverse Transcription Kit (Qiagen) was used to create cDNA from the purified RNA. qRT-PCR was performed using iQ SYBR Green Supermix (BioRad) with an iCycler iQ Real-Time Detection System (BioRad). qRT-PCR primers are listed in Supplemental Table A1. The measured C_T_ values were normalized using 16S rRNA, averaged, and wild type untreated condition was used to calculate the relative expression, ΔΔC_T_ value.

### Competitive Index

To determine if a competitive advantage resulted from high level macrolide resistance in *S. pneumoniae* competition assays were performed. Bacterial growth competitions were developed based on methods of Gupta et al. (2013). Overnight blood agar cultures were subcultured onto blood agar with or without supplementation with erythromycin at 0.5 μg/ml. Each strain was suspended in THY broth with or without erythromycin (0.5 μg/ml), grown to OD_600_ = 0.5–0.7 before dilution to OD_600_ = 0.050 in fresh media. Diluted cultures were mixed (1:1) for competition assays or grown independently as non-competition controls and grown to OD_600_ = 0.5–0.7 and diluted 200-fold in fresh media. Cultures were subcultured three times allowing the cultures to grow for approximately 50 generations. Sampling of cultures was performed to monitor growth phase by OD_600_. At *T* = 0 and each time the cultures reached late-log/stationary phase, culture aliquots were collected, serially diluted in phosphate-buffered saline, and plated on blood agar without selection (total culture density) and selective blood agar (one of the mutants): kanamycin 400 μg/ml, erythromycin 1 μg/ml, chloramphenicol 3.2 μg/ml, or tetracycline 2 μg/ml. The competitive index (CI) was calculated as CI = (mutant CFU_output_/wildtype CFU_output_)/(mutant CFU_input_/wildtype CFU_input_) and a CI < 1 indicates the mutant is less fit than then the wildtype.

### Statistical Analysis

Unpaired, two-tailed *t*-tests with 95% confidence intervals were performed using Prism^®^ 5 (GraphPad). For the growth competition experiments, the competitive index values of input were compared to the endpoint of 50 generations of growth.

## Results

### Macrolide Resistance in *S. pneumoniae* Due to Mega

In a large population-based collection of over 13,000 *S. pneumoniae*, Mega-containing macrolide resistant pneumococcal isolates were identified (section Materials and Methods). Table 3 shows the incidence of macrolide-resistant invasive pneumococcal disease (MR-IPD) 1999–2016 in Health District-3, Atlanta, GA, by macrolide resistance genotype across all ages before and after PCV7 introduction in 2000 and PCV13 introduction in 2010. Through 2016, 6 years after the introduction of PCV13 in the US, overall invasive pneumococcal disease was 6.94/100,000, macrolide resistance 2.0/100,000, *mef*(E)/*mel* resistance 1.11/100,000; and *erm*(B) resistance 0.67/100,000 (Table 3).

**Table 3:** Incidence of macrolide-resistant invasive pneumococcal disease (MR-IPD) 1999–2016 in Health District-3, Atlanta, GA, by macrolide resistance genotype across all ages.

Incidence	1999^a^	2003^a^	2010^b^	2013^b^	2016^c^
Overall IPD	29.4	14.02	11.51	8.90	6.94
Macrolide resistance	9.3	4.09	3.82	2.45	2.0
Mega [*mef*(E)/*mel*]	7.7	3.49	1.60	1.50	1.11
*erm*(B)	1.5	0.22	0.74	0.63	0.67
Dual resistance	ND	0.19	1.35	0.32	0.22

*Incidence reported as cases per 100,000 population. Dual resistance strains are those that contain mef(E)/mel and erm(B). (Rare MR-IPD strains that were not PCR positive for erm(B) or mef(E)/mel may be ribosomal mutations or an unknown mechanism.) ND, not determined. ^*a*^1999 prior to PCV7 (Stephens et al., 2005). ^*b*^2010 prior to PCV13 (Schroeder et al., 2017). ^*c*^This publication.*

**FIGURE 1:**
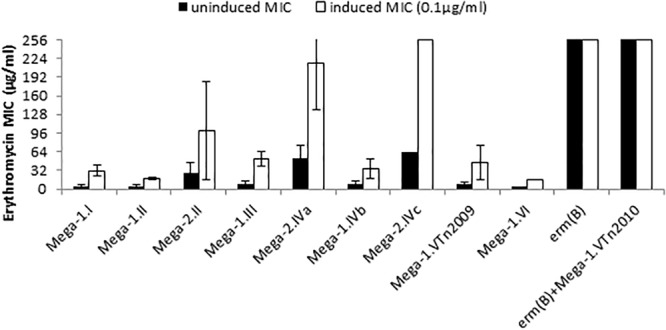
Macrolide resistance phenotypes and genotypes of *S. pneumoniae*. Erythromycin minimum inhibitory concentrations (MICs) were determined under uninduced conditions (black bars) and cultures induced with 0.1 μg/ml erythromycin (white bars). Each bar is the average MIC for a macrolide resistance genotype and strains in each group are detailed in Table 1.

Forty-four macrolide resistant *S. pneumoniae* isolates were chosen for further study, and demonstrated a wide range of resistance to erythromycin with MICs of 2 to > 256 μg/ml (Table 1 and Figure 1). Isolates with erythromycin MICs of 1–16 μg/ml were classified as “resistant” and we defined isolates with MICs > 16 μg/ml as “high-level resistant.” After overnight induction with subinhibitory erythromycin (0.1 μg/ml), the erythromycin MICs for these strains increased to 16 to > 256 μg/ml (Table 1 and Figure 1).

The relationship of MIC to differences in the *mef*(E)/*mel* intergenic region and the genomic sites of Mega insertion was assessed. Mega type 1 (Mega-1) and Mega type 2 (Mega-2) (Gay and Stephens, 2001), differentiated by a 99 bp insertion/deletion of the intergenic region between *mef*(E) and *mel*, did not contribute significantly to high-level macrolide resistance. Most Mega-1.II- and Mega-2.II-containing strains were found to have similar erythromycin and induced-erythromycin MICs (Figure 1). Strains with chromosomal insertion sites Mega-1.I, Mega-1.II, Mega-2.II, Mega-1.III, Mega-1.IVb, and Mega-1.VTn*2009* typically have uninduced erythromycin MICs 2–16 μg/ml (Table 1 and Figure 1), levels of macrolide resistance previously associated with Mega in *S. pneumoniae* (Gay and Stephens, 2001; Ambrose et al., 2005; Chancey et al., 2011), but with subinhibitory erythromycin induction MICs increased to 16–64 μg/ml (Table 1 and Figure 1). These isolates were also susceptible to clindamycin and thus have an M-phenotype. The newly described Mega-1.novel insertion (Chancey et al., 2015a), herein named Mega-1.VI, exhibited an MIC of 4 μg/ml and an induced-erythromycin MIC of 16 μg/ml.

### Mega Only-Containing *S. pneumoniae* With High-Level Macrolide Resistance

*S. pneumoniae* containing Mega-2.IVa or Mega-2.IVc insertions exhibited intrinsic high-level macrolide resistance, with uninduced erythromycin MICs of >18–256 μg/ml erythromycin and induced MICs of >96 to > 256 μg/ml erythromycin (Table 1 and Figure 1). The Mega-2.IVa and Mega-2.IVc isolates were found to be clindamycin susceptible (M phenotype). No other macrolide resistant determinants were found in these isolates, and mutation of the Mega element resulted in erythromycin sensitivity with MICs of 0.125 μg/ml (Table 2).

### Macrolide Resistance in *S. pneumoniae* Containing *erm*(B) and *mef*(E)/*mel*

As anticipated, Mega-1.V Tn*2010* isolates containing both *erm*(B) and Mega (Del Grosso et al., 2006) had uninduced erythromycin MICs of >256 μg/ml and were clindamycin resistant (MLS_B_ phenotype). To better understand the relative roles of *mef*(E)/*mel* and *erm*(B) in high-level macrolide resistant Tn*2010*-containing *S. pneumoniae*, isogenic deletion mutations of *erm*(B) or *mef*(E)/*mel* or both were made in strain GA44288, an invasive pneumococcal disease isolate (Chancey et al., 2015a). The deletion of *mef*(E)/*mel* from GA44288, strain MS41 [Tn*2010*Δ*mef*(E)/*mel*], had no effect on erythromycin or clindamycin resistance, as MS41 remained highly resistant to erythromycin (MIC of > 256 μg/ml) (Table 2). The deletion of *erm*(B) in GA44288 generated the mutant MS32 [Tn*2010*Δ*erm*(B)], which displayed an M phenotype with an erythromycin MIC of 8 μg/ml, an induced erythromycin MIC of 64 μg/ml (Table 2), and susceptibility to clindamycin. These MIC data for MS32 are consistent with the majority of Mega-only containing isolates (Table 1 and Figure 1). The deletion of the dual macrolide resistance determinants [*mef*(E)/*mel* and *erm*(B)] in GA44288 was designated MS42 [Tn*2010* Δ*erm*(B)Δ*mef*(E)/*mel*]. MS42 was susceptible to erythromycin (MIC 0.125 μg/ml) and clindamycin (Table 2). These data confirmed *erm*(B) and *mef*(E)/*mel* as the only macrolide resistance determinants in GA44288 and that the high-level macrolide resistance of Tn*2010*-containing *S. pneumoniae* was due to the presence of *erm*(B).

In *S. pneumoniae*, the expression of *mef*(E) and *mel* is controlled through transcriptional attenuation (Chancey et al., 2015b). Macrolide-induced ribosomal stalling results in deattenuation of *mef*(E)/*mel* to produce full-length polycistronic transcripts (Chancey et al., 2015b). To determine if *mef*(E)/*mel* was expressed in the presence of *erm*(B), *mef*(E) expression was measured by qRT-PCR from GA44288 after a 15 min exposure to erythromycin. The expression of *mef*(E) was dose-dependent, and 0.5 μg/ml erythromycin was sufficient to induce similar *mef*(E) expression in both the wild type and the *erm*(B) deletion mutant (data not shown). Thus, in isolates with both *erm*(B) and Mega, *mef*(E)/*mel* expression was induced by erythromycin and shown by the resistance data to result in a functional efflux pump/ribosomal protection protein.

### *mef*(E)/*mel* Alone Was Responsible for High-Level Macrolide Resistance in Mega-2.IVa- and Mega-2.IVc-Containing *S. pneumoniae*

The molecular basis for Mega-2.IVa and Mega-2.IVc isolates with high-level macrolide resistance but the M phenotype was further assessed. In the high-level macrolide-resistant strain GA16242 with a Mega-2.IVa insertion (uninduced erythromycin MIC of 64 μg/ml) *mef*(E)/*mel* was deleted to create TS9001. TS9001 was susceptible to erythromycin at 0.125 μg/ml (Table 2). Similarly, the deletion of *mef*(E)/*mel* from the high-level macrolide-resistant Mega-2.IVc strain GA17545 (uninduced erythromycin MIC of 64 μg/ml) resulted in susceptibility to macrolides as the erythromycin MIC of the mutant designated XZ8012-5 was 0.19 μg/ml (Table 2). Thus, *mef*(E)/*mel* alone in *S. pneumoniae* Mega-2.IVa and Mega-2.IVc isolates was responsible for high-level macrolide resistance.

To further confirm that the Mega class IVa and IVc insertions resulted in high-level macrolide resistance and determine whether the high-level macrolide resistance phenotype was transferable, the Mega-2.IVa insertion was transformed into the erythromycin susceptible strain NP112 (MIC 0.19 μg/ml). The resulting NP112 Mega-2.IVa isolate (designated MS23) demonstrated high-level macrolide resistance with an erythromycin MIC of 32 μg/ml and was inducible up to >256 μg/ml (Table 2). The transfer of Mega-2.IVa included the adjacent IS*Smi*2 element and recreated the pneumococcal pathogenicity island (PPI-1) deletion found in Mega-2.IVa isolates (Chancey et al., 2015a). Deletion of *mef*(E)/*mel* from MS23 (designated MS30) restored macrolide susceptibility (Table 2). Mega-mediated high-level macrolide resistance was also transferred to the GA17457 Δ*mef*(E)/*mel* deletion strain (XZ8009). After transformation of XZ8009 with the Mega-2.IVa insertion (designated MS27), MS27 was found to have an erythromycin MIC of 32 μg/ml inducible up to >256 μg/ml (Table 2).

### High-Level Macrolide Resistance, Regardless of Mechanism, Provided a Competitive Advantage for Growth During Exposure to Erythromycin

**FIGURE 2:**
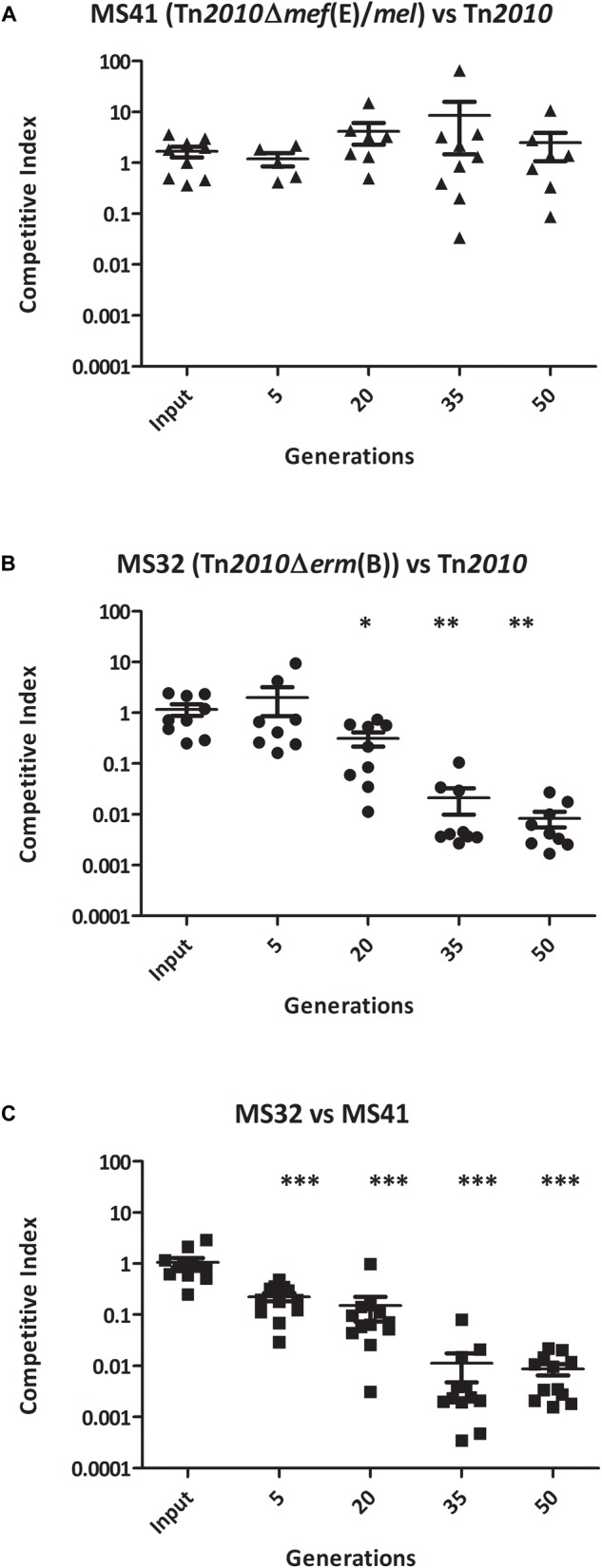
The competitive index of isogenic GA44288 Tn*2010* mutants grown *in vitro* with erythromycin (0.5 μg/ml) for approximately 50 generations: **(A)** MS41 [Tn*2010*Δ*mef*(E)/*mel*] versus Tn*2010*, **(B)** MS32 [Tn*2010*Δ*erm*(B)] versus Tn*2010*, and **(C)** MS32 [Tn*2010*Δ*erm*(B)] versus MS41 [Tn*2010*Δ*mef*(E)/*mel*]. ^∗^*p* < 0.05, ^∗∗^*p* < 0.01, ^∗∗∗^*p* < 0.001.

To determine whether *erm*(B) and/or *mef*(E)/*mel* in *S. pneumoniae* provided a competitive advantage for growth during exposure to erythromycin, competitive assays using the clinical isolate GA44288 containing *erm*(B) and *mef*(E)/*mel* on Tn*2010*, and the strains with mutations in these genes (Table 2) were performed. Erythromycin-induced cultures of wild type strain GA44288 [*erm*(B) and *mef*(E)/*mel* on Tn*2010*] and the isogenic mutants MS32 [Δ*erm*(B), *mef*(E)/*mel*] and MS41 [*erm*(B), Δ*mef*(E)/*mel*)] (Table 2) were used in an *in vitro* competitive index (1:1 ratio). The concentration of erythromycin used is known to be achieved in human serum during treatment (0.5 μg/ml) (Metzler et al., 2013). No significant difference in growth was observed between MS41 [*erm*(B), Δ*mef*(E)/*mel*] and the wild type strain GA44288 [*erm*(B), *mef*(E)/*mel*] in this assay (Figure 2A, *p* = 0.5460). This suggested that *mef*(E)/*mel* did not provide a growth advantage to an *erm*(B)-containing strain during exposure to erythromycin. When MS32 [Δ*erm*(B), *mef*(E)/*mel*] was competed with GA44288 [wild type, Tn*2010* with *erm*(B)*, mef*(E)*mel*] the competitive index decreased to approximately 0.01 after 50 generations (Figure 2B). These data indicated a significant growth advantage due to the high-level resistance encoded by *erm*(B) for GA44288 during exposure to erythromycin (*p* = 0.0012). Similarly, the competitive index of MS32 [Δ*erm*(B), *mef*(E)/*mel*] versus MS41 [*erm*(B), Δ*mef*(E)/*mel*] dropped to approximately 0.01 by the endpoint 50 generations (Figure 2C, *p* < 0.0001), again confirming the importance of *erm*(B)-induced high-level macrolide resistance during exposure to erythromycin. When these experiments were performed without erythromycin, the competitive indexes were at ∼1 throughout the course of the experiments (Supplemental Figure A1). Thus, in each of the erythromycin-induced competitive index experiments, an active *erm*(B)-containing strain (GA44288 and MS41) had a competitive advantage over the *erm*(B) deletion stain (Figure 2). The data suggest that *erm*(B) provides a growth advantage when *S. pneumoniae* is exposed to treatment-level concentrations of erythromycin.

**FIGURE 3:**
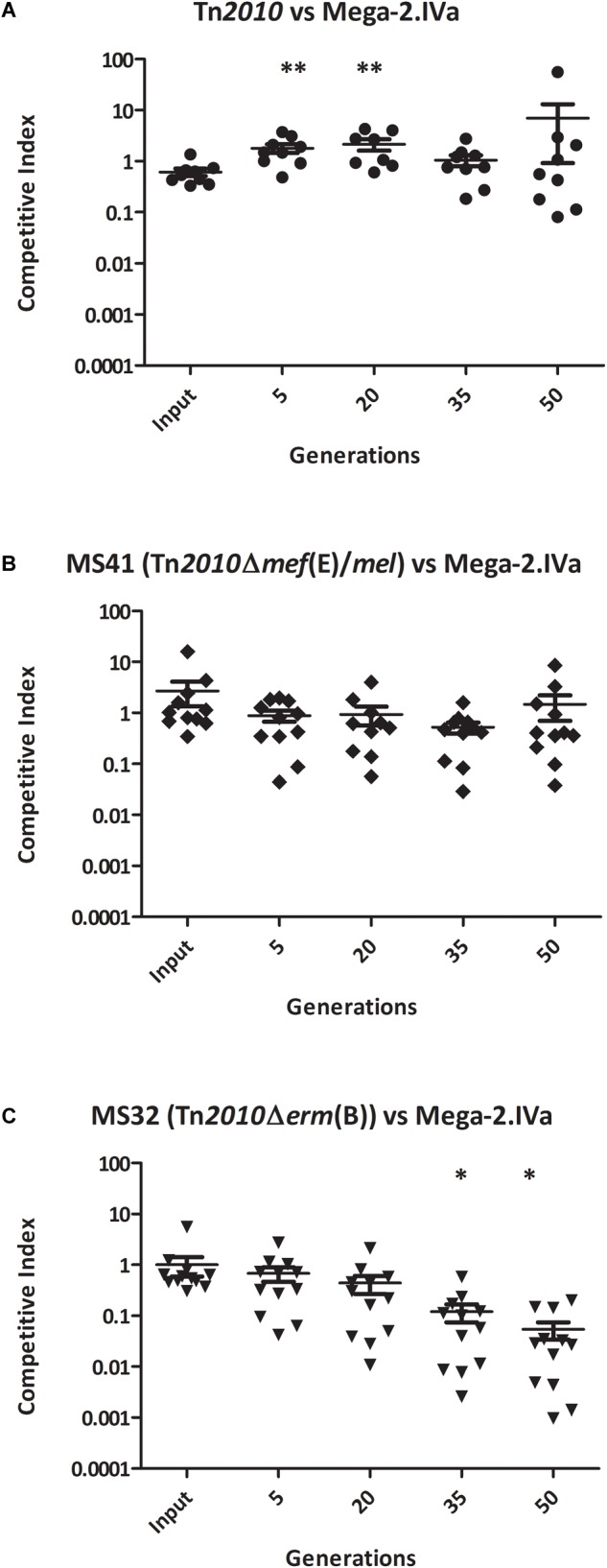
The competitive index of high-level macrolide resistance strains with distinct mechanisms [*erm*(B) and Mega-2.Iva] *in vitro* with erythromycin (0.5 μg/ml) grown for approximately 50 generations: **(A)** GA44288 [*erm*(B)-and *(*mef*(E)/*mel*-containing] versus GA16242 (Mega-2.IVa-containing), **(B)** MS41 [GA44288 Δ*mef*(E)/*mel, erm*(B)-containing] versus GA16242 (Mega-2.IVa-containing), and **(C)** MS32 [GA44288 Δ*erm*(B)] versus GA16242 (Mega-2.IVa-containing). ^∗^*p* < 0.05, ^∗∗^*p* < 0.01.)*

To determine whether the competitive advantage for growth of *erm*(B) during erythromycin exposure was due Erm(B)-mediated ribosomal methylation or to the high-level macrolide resistance of the *erm*(B)-containing strains, the competitive index assay was performed with the GA44288 isogenic strains in competition with GA16242, the Mega-2.IVa strain that produced high-level macrolide resistance due only to the presence of *mef*(E)/*mel* (Figure 1 and Table 2). The competitive index for GA44288 versus GA16242 remained ∼1 throughout the course of the experiments (Figure 3A, *p* = 0.3088). The competitive index for MS41 [*erm*(B), Δ*mef*(E)/*mel*] versus GA16242 [*mel*(E)/*mef*, Mega-2.IVa] also did not change throughout the experiments (Figure 3B, *p* = 0.4397). These data suggest that high-level macrolide resistance and not Erm(B)-mediated ribosomal methylation was responsible for the growth advantage in erythromycin. Finally, we assayed MS32 [GA44288 Δ*erm*(B), *mel*(E)/*mef*] with GA16242 and found the competitive index decreased below 0.1 after 50 generations of growth (Figure 3C, *p* = 0.0316). Thus, both high-level macrolide resistance strains (GA44288 and GA16242), albeit generated by different mechanisms, provided the growth competitive advantage during exposure to erythromycin.

## Discussion

In the United States, the rapid rise of macrolide resistance (includes resistance to erythromycin, azithromycin, clarithromycin and other 14- and 15-membered macrolides) in *S. pneumoniae* throughout the 1990s was due to the horizontal transfer and clonal expansion of stains containing the *mef*(E)/*mel* encoded efflux pump and ribosomal protection protein. The *mef*(E)/*mel* operon is found in the pneumococcal genome on the 5.4–5.5 kb Mega element (Stephens et al., 2005; Rudolph et al., 2013). Mega-mediated pneumococcal macrolide resistance has generally been associated with lower levels of macrolide MICs (1–16 μg/ml to erythromycin) compared to high levels of macrolide resistance observed for *erm*(B) (usually > 256 μg/ml to erythromycin) (Rudolph et al., 2013). The use of *in vitro* assays to query MIC values may be a limitation, however, macrolide resistance in the pneumococcus, caused by either *erm*(B) or *mef*(E)/*mel*-containing isolates, has been linked to treatment failures for lower respiratory tract infections and bacteremia (Klugman, 2002; Lonks et al., 2002; Schentag et al., 2007; Zähner et al., 2010). In one study by Gonzalez et al. (2004) 10/11 isolates from azithromycin treatment failures, who did not have underlying medical conditions, had the *erm*(B) (two patients) or Mega (eight patients) phenotype and three of the six available Mega-containing isolates had MICs ≥ 16 μg/ml.

We previously defined the genetic basis for the resistance mediated by the *mef(*E)/*mel*-containing Mega element in *S. pneumoniae*, demonstrated that *mef*(E) and *mel* are inducible by most 14- and 15-membered macrolides (including erythromycin, clarithromycin, and azithromycin) and by antimicrobial peptides, defined the Mega element that contains *mef*(E)/*mel*, identified the mechanism of macrolide induction of *mef*(E)/*mel*, demonstrated Mega’s relationship to conjugative transposons, and mapped the locations of Mega in the pneumococcal genome (Gay and Stephens, 2001; Zähner et al., 2010; Chancey et al., 2011, 2015a,b). Mega elements are either type 1 or 2, distinguished by a 99 bp insertion/deletion (Mega-1 at 5.5 kb and Mega-2 at 5.4 kb) (Gay and Stephens, 2001). Mega is found in the pneumococcal genome in six distinct sites, termed Mega classes (Chancey et al., 2015a). Insertion sites numbered I-IV were originally described (Gay and Stephens, 2001). When inserted into *orf6* of Tn*916*-like elements, Mega is classified as insertion site V (Del Grosso et al., 2004, 2006, 2007). Recently, we reported a new chromosomal insertion site, VI (Chancey et al., 2015a). Mega class IV has been further classified into three subclasses: Mega-2.IVa, Mega-1.IVb, and Mega-2.IVc all of which are upstream of the Pneumococcal Pathogenicity Island-1 (PPI-1) (Chancey et al., 2015a).

High-level macrolide resistance (MIC > 16 μg/ml) due to Mega has been observed in clinical isolates (Gonzalez et al., 2004; Rudolph et al., 2013) but has not been not characterized at a genomic level. We found high-level macrolide resistance in *S. pneumoniae* due to Mega related to specific genomic insertions Mega-2.IVa and Mega-2.IVc. We also have recent evidence of M-phenotype high-level macrolide resistance in Mega-2.II strains (unpublished data). Most Mega-2.IVa and Mega-2.IVc have high-level macrolide resistance while the Mega-1.IVb insertions have MICs similar to the other Mega insertion sites (Chancey et al., 2015a). The molecular mechanism of high-level macrolide resistance due to efflux and ribosomal protection in Mega-2.IVa/c and the newly discovered Mega-2.II isolates is not understood and is a limitation of this study, but is currently under investigation. One possibility is that high level macrolide resistance is related to genomic “trans” factors favoring higher expression the *mef/mel* operon, and this is associated with specific clonal complexes such as ST156 or ST15. Baseline MICs and *mef/mel* operon expression are elevated in strains with high level resistance. The 99-bp insertion in the intergenic region between *mef*(E) and *mel*, the distinguishing characteristic between Mega-1 from Mega-2, does not appear to significantly contribute to high-level macrolide resistance.

*S. pneumoniae* containing both macrolide resistance genetic elements, *mef*(E)/*mel* and *erm*(B), were first noted in the late-1990s from the US, Japan, and South Africa (Corso et al., 1998; Luna et al., 1999; Nishijima et al., 1999; McGee et al., 2001). Many of these strains were initially noted to belong to a single 19F multidrug resistant clone and subsequent work identified this clone as lineage Taiwan^19F^-14, or PMEN14 (McGee et al., 2001; Croucher et al., 2014). Dual element macrolide-resistant serotypes, especially 19A *S. pneumoniae* belonging to CC320 (formerly CC271) a PMEN14 lineage (Del Grosso et al., 2007; Bowers et al., 2012; Chancey et al., 2015a), have increased steadily in the US and worldwide (Moore et al., 2008; Mahjoub-Messai et al., 2009; Schroeder et al., 2017). For example, clonal expansion of dual macrolide-resistant 19A *S. pneumoniae* expanded dramatically in the US after the introduction of the pneumococcal conjugate vaccine, PCV7 (Moore et al., 2008). We documented the decline, 2010–2013 of serotype 19A (CC 320) with dual element macrolide resistance mechanisms following the introduction of PCV13 in 2010 (Schroeder et al., 2017). As part of this study, we analyzed our 2013–2016 population data set and isolate collection to further understand the impact of PCV13 on macrolide resistance in the population and the continued importance of the dual element resistance strains.

Through 2016, 6 years after the introduction of PCV13, the incidence of invasive pneumococcal disease has declined but the percent of these isolates that are macrolide resistance has remained at ∼30%. The *erm*(B)/*mef*(E)/*mel* dual element resistance strains continue to circulate with an incidence of 0.22/100,000 in the population (Table 3). These dual element resistance strains remain primarily 19A (CC320). While the samples for this study were collected from a defined geographic area, this potential limitation is moderated by the persistence of dual element resistant strains which were also observed throughout the US in 2016.

*S. pneumoniae* isolates with the dual element macrolide resistance genotype are carried on the mobile Tn*2010* and are multidrug resistant (McGee et al., 2001; Bowers et al., 2012; Chancey et al., 2015a). Tn*2010* is a large 26⋅4-kb element with Mega [*mef*(E)/*mel*] and Tn*917* [*erm*(B)] inserted at two distinct sites into a Tn*916*-like conjugative transposon (Del Grosso et al., 2007). A possible origin of Tn*2010* is via homologous recombination of a Tn*2009* (Mega-containing) strain acquiring a Tn*6002* fragment with *erm*(B) flanked by Tn*916 orf20*, or a Tn*6002* [*erm*(B)-containing] strain acquiring a *Tn2009* fragment with Mega flanked by Tn*916 orf*6 (Chancey et al., 2015a; Schroeder and Stephens, 2016). This dual element macrolide resistance genotype results in an MLS_B_ phenotype with high-level macrolide resistance. These elements are not only able to transmit between co-resident serotypes of *S. pneumoniae* within the nasopharyngeal niche, but also have the possibility of transmitting to other naturally competent bacteria present in the nasopharynx, such as the clinically relevant *Haemophilus influenzae* (Lattar et al., 2018).

In this study, we found both *mef*(E)/*mel* and *erm*(B) are expressed and functional in the Tn*2010* background. This indicates the co-expression of macrolide determinants in these strains. While high-level macrolide resistance of these *S. pneumoniae* isolates with the dual element macrolide resistance genotype was dominated by *erm*(B), which also confers resistance to lincosamides and streptogramin B, *mef*(E)/*mel* may provide other selective advantages. The presence of *mef*(E)/*mel* has been found to enhance resistance to the human antimicrobial peptide LL-37 (Zähner et al., 2010). Thus, selective pressure for the acquisition and maintenance of both *mef*(E)/*mel* and *erm*(B) in *S. pneumoniae* may be encountered in human colonization and disease.

Antibiotic resistance determinants are often inducible and provide a selective advantage over non-resistant organisms in an antibiotic-containing environment. However, the expression of these determinants can be associated with a fitness cost (Andersson and Hughes, 2010). In the pneumococcus, *erm*(B) commonly is inducible and tightly regulated through translational attenuation (Montanari et al., 2001; Min et al., 2008). The *mef*(E)/*mel* operon is also tightly regulated but through transcriptional attenuation (Chancey et al., 2015b). While the expression of *erm*(B) in *S. pneumoniae* by a partial attenuator deletion did not cause a growth defect when the strain was grown *in vitro* as a pure culture (Wolter et al., 2008), in *Staphylococcus aureus* deregulation of *erm*(C), a homolog of *erm*(B), results in increased expression of a subset of the proteome that causes a 10-fold fitness defect *in vitro* (Gupta et al., 2013).

We found that *erm*(B) in *S. pneumoniae* did not cause a growth defect and provided a competitive advantage up to 100-fold over a lower-level resistant Mega-containing strain during growth in a macrolide containing environment. Insertions of *mef*(E)/*mel* causing high-level macrolide resistance when used in the competitive growth assays also provided a substantial growth fitness increase over the lower-level resistant *mef*(E)/*mel* phenotype when exposed to erythromycin. With a low fitness burden for maintenance of *mef*(E)/*mel* and/or *erm*(B), these determinants are unlikely to be lost from the pneumococcal population even with reduced use of antibiotics in clinical settings (Andersson and Hughes, 2010). Active efflux of the macrolide into the environment by Mega high-level resistant strains growing at high density may provide a further fitness advantage over low-level resistant strains.

An effective measure to date in combating antibiotic-resistant pneumococcal infections has been the introduction of pneumococcal conjugate vaccines (Stephens et al., 2005). These vaccines provide individual protection for vaccinated individuals and reduce transmission of vaccine serotypes leading to herd protection for unvaccinated individuals in the same population (Rodgers and Klugman, 2011). The targeted vaccine serotypes have often had the highest rates of antibiotic resistance determinants (Stephens et al., 2005). However, as shown in the recent surveillance data macrolide resistance in genetic elements capable of horizontal transfer to new serotypes continues to persist in invasive *S. pneumoniae*.

In summary, likely driven by macrolide use in the population, high-level macrolide resistance in *S. pneumoniae* due to the Mega element alone has emerged, specifically in the context of the Mega-2.IVa or Mega-2.IVc and Mega-2.II genomic insertions. In addition, Tn*2010*-containing *S. pneumoniae* isolates that carry both *erm*(B) and *mef*(E)/*mel* have rapidly expanded with both resistant determinants functional in these strains. High-level macrolide resistance regardless of mechanism provides a competitive advantage for growth during exposure to macrolides.

## Author Contributions

MS, SC, and DS designed the study. MS and SL collected the data. MS, SL, SC, and DS performed the analysis. MS, SL, and DS wrote the manuscript.

## Conflict of Interest Statement

The authors declare that the research was conducted in the absence of any commercial or financial relationships that could be construed as a potential conflict of interest.
