# Supplementary figures and images for: High-Level Macrolide Resistance Due to the Mega Element [mef(E)/mel] in Streptococcus pneumoniae
Source: Front Microbiol. 2019 Apr 24;10:868. doi: 10.3389/fmicb.2019.00868 (PMC6491947; doi:10.3389/fmicb.2019.00868)

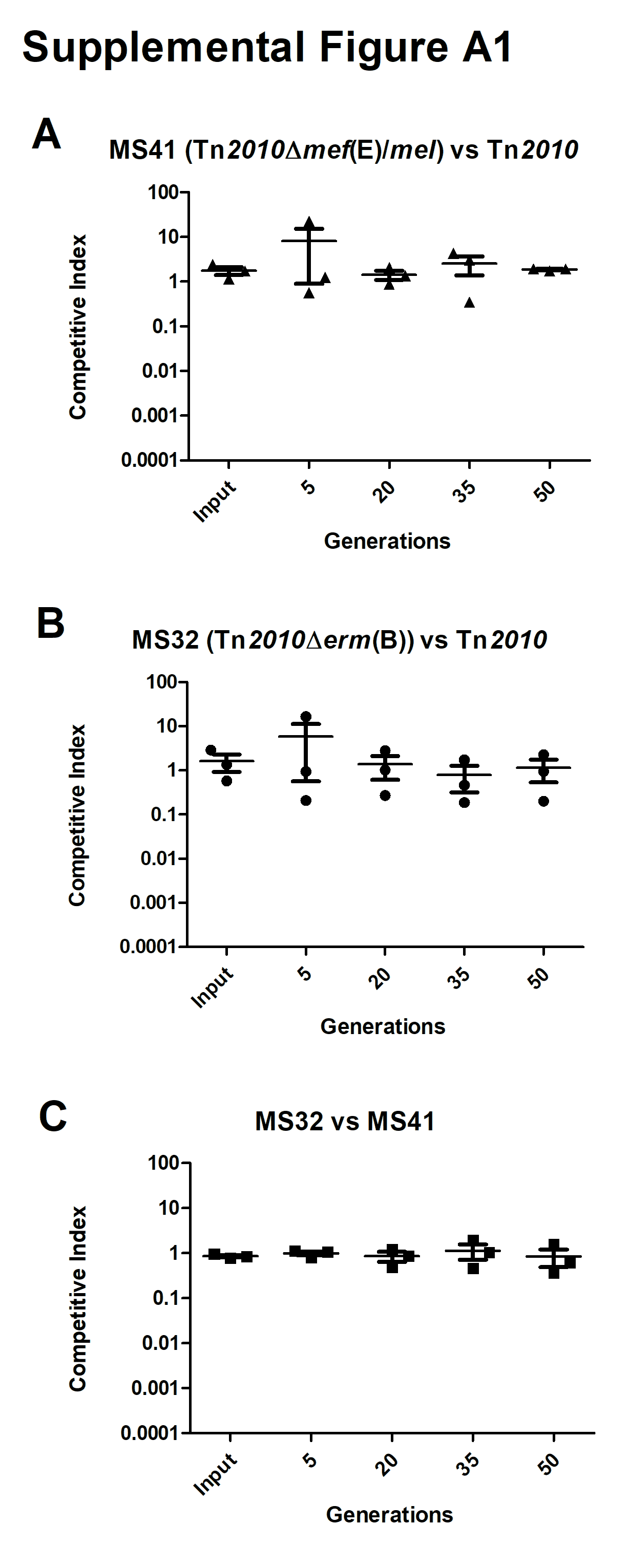

Supplement: Supplementary file 1 [file Image_1.tif]
