# Supplementary material for: High-Level Macrolide Resistance Due to the Mega Element [mef(E)/mel] in Streptococcus pneumoniae
Source: Front Microbiol. 2019 Apr 24;10:868. doi: 10.3389/fmicb.2019.00868 (PMC6491947; doi:10.3389/fmicb.2019.00868)
Supplement: Supplementary file 2 [file Data_Sheet_1.docx]

# SUPPLEMENTAL MATERIALS

**Supplemental Table A1.** Primers used in the study.

| Cloning Primers | Sequence (5’ to 3’) | Restriction Site^a^ |
| --- | --- | --- |
| SC173 | CCAGAGATTGTGTCTGTCATGC |  |
| SC251 | GCTAGTCACTAGACGGTTTAGACC |  |
| SC10 | acttgtcaatcacggacagc |  |
| SC70 | GCAAGTTGCTGCTAGACACTG |  |
| SC125 | agcaagagagtaccaggatac |  |
| kanA | cttagcaggagacattccttccg |  |
| kanC | gtggtatgacattgccttctgcg |  |
| MS27 | AGCGTGCCTATTATGCAGTC |  |
| MS28 | TCGCCAAAATGCTGTTGATC |  |
| MS34 | ATTAGGATCCTATCAATCGGTATATCCGTT | BamHI |
| MS35 | CGTGTAACTTTCCAAATTTATCTAGACTTAAGTTTGCTTCTAAGTC | XbaI |
| MS36 | GACTTAGAAGCAAACTTAAGTCTAGATAAATTTGGAAAGTTACACG | XbaI |
| MS37 | TAATCTGCAGACTTACCAAGATATCACGAA | PstI |
| MS53 | TATATCTAGACAGGACAATAACCTTATAGC | XbaI |
| MS54 | TATATCTAGACCAACTTACTTCTGACAAC | XbaI |
| MS63 | TATAGGATCCCATTTTGATAAAAACTACAACAGG | BamHI |
| MS64 | TATACTGCAGAGCCTTGATTGCAAGGC | PstI |
| MS69 | GCTTATCGATACCGTCGAATTTAAGGTAGTCGCTGG |  |
| MS70 | CCAGCGACTACCTTAAATTCGACGGTATCGATAAGC |  |
| MS71 | GGAAGTATGAGTCTCATTCCAGTTAGTGACATTAGAAAACCG |  |
| MS72 | CGGTTTTCTAATGTCACTAACTGGAATGAGACTCATACTTCC |  |
| qRT-PCR Primers | **Sequence (5’ to 3’)** | **Amplified Locus** |
| q16S_F2 | CCAGATGGACCTGCGTTGTAT | 16S rRNA |
| q16S_R2 | TCCGTCCATTGCCGAAGATT | 16S rRNA |
| qmef_F3 | gtattcccgaaacggctaaactg | *mef*(E) |
| qmef_R3 | tggaacgcctgtgcatatttc | *mef*(E) |
| qmel_F2 | ttctgcaccgactatagggtatgg | *mel* |
| qmel_R2 | aaaccctagagcacaggattgc | *mel* |
| qerm_F2 | ccgaacactagggttgctctt | *erm*(B) |
| qerm_R2 | tgtggtatggcgggtaagtt | *erm*(B) |

^a^ Restriction sites are underlined.
